# Reconfigurable second-order optical all-pass filter

**DOI:** 10.1515/nanoph-2022-0140

**Published:** 2022-05-27

**Authors:** Yu Chen, Lu Xu, WeiJun Jiang, Lin Wang, Shuai Cui, Yu Yu, Yuan Yu, Xinliang Zhang

**Affiliations:** Wuhan National Laboratory for Optoelectronics and School of Optical and Electronic Information, Huazhong University of Science and Technology, Wuhan 430074, China; Optics Valley Laboratory, Wuhan 430074, China

**Keywords:** all-pass filter, microwave photonic phase shifter, silicon photonics, variable time delay

## Abstract

The optical all-pass filter (APF), which exhibits a constant amplitude response and a variable phase response, is a key to manipulating the optical phase without inducing signal amplitude distortion. High-order APFs are significantly demanded because they can afford large time delays and phase shifts. However, to date, only first-order APFs based on lossy waveguides have been reported. Although high-order APFs can be simply obtained by cascading multiple first-order APFs, the complexity and size are increased. To solve this problem, we propose and demonstrate a second-order APF using Mach–Zehnder interferometer-assisted microring resonators. The device is fabricated based on a silicon-on-insulator platform. Based on the second-order APF, an adjustable time delay between 553 and 948 ps is obtained, and the corresponding amplitude variation is less than 1.7 dB. Meanwhile, a microwave photonic phase shifter is also obtained based on the APF. The microwave phase shift can be adjusted from 0 to 3.27*π*, with an RF power variation within 2.4 dB. Additionally, the second-order APF can be reconfigured to a first-order APF, which significantly enhances its flexibility. The reconfigured first-order APF can realize an adjustable time delay between 257 and 429 ps, and the amplitude variation is less than 0.9 dB. The proposed high-order APF provides a novel approach to manipulating optical signals.

## 1 Introduction

Recently, silicon-on-insulator (SOI)-based optical signal processing devices have attracted great interest because of their inherent advantages in size, weight, power, and cost (SWaP-C) [1–3]. Optical filters are of fundamental importance in eliminating noise or extracting signals via amplitude manipulation [4–6]. In contrast to filters exploiting varied amplitude responses, all-pass filters (APFs) have a constant amplitude response and a variable phase response, which is especially suitable for signal phase manipulation without introducing amplitude distortion [7], such as in adjustable delay lines [8, 9], microwave photonic phase shifters [10–12], optical add-drop multiplexers [13], optical dispersion compensators [14, 15], and Hilbert transformers [16].

To achieve an APF, the zero locations of the system function are mirror images about the unit circle from the pole locations [7]. In recent decades, APFs have been developed based on ideal lossless conditions, where the waveguide loss and coupling loss are omitted. However, lossless conditions cannot be obtained in practical passive waveguides, and there is always amplitude variation in the achieved APFs. To eliminate the amplitude variation induced by loss, several approaches have been proposed. First, a method called self-compensation of loss was proposed, and an APF was obtained [17]. Then, obtaining an APF by using optical interference between the outputs of an all-pass microring resonator and a straight waveguide was also proposed [18]. The obtained APF exhibits ultralow amplitude variation and the APF-based variable optical delay line and microwave photonic phase shifter also exhibit ultralow amplitude distortion. Notably, both achieved APFs are first-order types. Hence, based on the first-order APF, the achieved microwave photonic phase shift is less than 2*π*, and the achieved maximal time delay is also limited. To promote the system performance, high-order APFs are desired. Although high-order APFs have been proposed to achieve a large time delay by cascading multiple MRRs [7, 14, 19, 20], amplitude variation exists in the phase shift region of the APF and signal distortion is consequently induced. Because these high order APFs can only be obtained for lossless waveguides. Cascading first-order APFs is a direct approach to acquire a high-order APF. However, the insertion loss, size and complexity are multiplied as the order of the APF increases.

In this paper, we propose and demonstrate a second-order APF by simply cascading a microring resonator (MRR) and a structure of a first-order APF. Compared with the second-order APF obtained by simply cascading two first-order APFs, the novel structure has superiorities in size, loss and simplicity, which is more obvious when extended to higher-order APFs. The device is fabricated based on an SOI wafer. Notably, the second-order APF can also be reconfigured to a first-order APF, which enhances the flexibility in applications. APF-based variable optical delay line and microwave photonic phase shifter are also demonstrated. The time delay and the microwave phase shift based on the second-order APF can be continuously adjusted from 550 to 948 ps and from 0 to 3.27*π*, respectively. One advantage of the proposed approach is that the topology of the proposed second-order APF can be easily extended to higher-order APFs (see Supplementary Material Section 1). The proposed high-order APF provides another dimension for manipulating optical

signals in addition to the amplitude response of optical filters, which means that pure phase manipulation can be achieved without resulting in amplitude distortion.

## 2 Device design and principle

In an optical filter, the optical interference can be used to adjust the zero locations without changing the pole locations. In our design, two cascaded Mach–Zehnder interferometers (MZIs) are used to arbitrarily adjust the zero locations of the device, and two MRRs provide the phase shift and time delay. Notably, to realize a broadband second-order APF, the two MRRs should have the same free spectral range (FSR). Therefore, the two MRRs are designed with equal cavity lengths.

**Figure 1:**
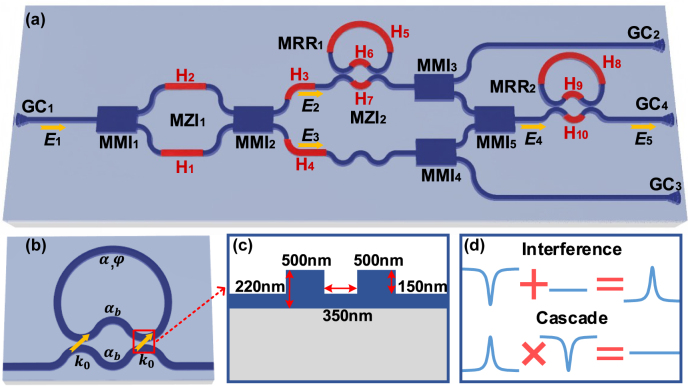
Principle. (a) Schematic diagram of the proposed APF. MRR, microring resonator; MMI, multimode interferometer; MZI, Mach–Zehnder interferometer; GC, grating coupler. The yellow arrows represent the propagation of the optical field. (b) Schematic diagram of the MRR with an adjustable coupling coefficient based on the MZI coupler. (c) Cross section of the coupling region. (d) Depiction of the principle of the second-order APF.

Figure 1(a) shows a schematic diagram of the proposed second-order APF, which was designed based on an SOI wafer with a top layer of 220 nm (see in Supplementary Material Section 2). The optical signal, which is coupled into the device by a grating coupler (GC_1_), is denoted as *E*_1_. The propagation direction of the optical signal is denoted by the yellow arrow in Figure 1(a). Then, a multimode interferometer (MMI_1_) equally divides the optical signal into the two arms of MZI_1_. The phase difference between the two arms of MZI_1_ can be changed by adjusting the electric power applied to H_1_ or H_2_. After phase adjustment, the two optical beams are combined and then redivided into two parts by MMI_2_. The optical field of the upper (*E*_2_) and lower (*E*_3_) arms of MZI_2_ are described as

(1) $$\underline{\left[\begin{array}{c} E_{2}\\ E_{3} \end{array}\right]=\frac{\sqrt{2}}{2}\left[\begin{array}{cc} 1 & j\\ j & 1 \end{array}\right]\times \left[\begin{array}{cc} 1 & 0\\ 0 & {\text{e}}^{j\phi_{1}} \end{array}\right]\times \frac{\sqrt{2}}{2}\left[\begin{array}{c} 1\\ 1 \end{array}\right]E_{1}=\frac{1}{2}\left[\begin{array}{c} 1+j{\text{e}}^{j\phi_{1}}\\ j+{\text{e}}^{j\phi_{1}} \end{array}\right]E_{1},}$$

where *ϕ*_1_ is the phase difference between the two arms of MZI_1_. The amplitude splitting ratio between the lower and upper arms of MZI_2_ *x* is expressed as

(2) $$\underline{x=\frac{E_{3}}{E_{2}}=\frac{j+{\text{e}}^{j\phi_{1}}}{1+j{\text{e}}^{j\phi_{1}}}\text{. }}$$

Then, the upper part of the light is coupled with an MRR (MRR_1_), and the lower part is sent to the lower arm of MZI_2_. To eliminate the coupling deviation from the designed value caused by fabrication error (see in Supplementary Material Section 3), the coupling between MRR_1_ and the upper arm of MZI_2_ is designed with a balanced MZI structure, and the coupling ratio can be changed by adjusting the electric power applied to microheater H_6_ or H_7_. Microheaters H_3_ and H_4_ are used to adjust the phase difference between the two arms of MZI_2_. Notably, MMI_3_ and MMI_4_ are designed to extract the transmissions of the two arms of MZI_2_, and GC_2_ and GC_3_ are designed as the monitor ports. The optical signals in the two arms of MZI_2_ are combined in MMI_5_. Omitting the optical loss caused by MMI_3_ and MMI_4_, the optical field of the combined optical signal *E*_4_ is expressed as

(3) $$\underline{E_{4}=\frac{\sqrt{2}}{2}\left[\begin{array}{cc} 1 & 1 \end{array}\right]\times \left[\begin{array}{cc} H_{\text{MR}{\text{R}}_{1}}\left(f\right) & 0\\ 0 & {\text{e}}^{j\phi_{2}} \end{array}\right]\times \frac{1}{2}\left[\begin{array}{c} E_{2}\\ E_{3} \end{array}\right]=\frac{\sqrt{2}}{4}\left(1+j{\text{e}}^{j\phi_{1}}\right)\left(\frac{t_{1}-\alpha {\text{e}}^{j\varphi }}{1-\alpha t_{1}{\text{e}}^{j\varphi }}+{\text{e}}^{j\phi_{2}}x\right)E_{1},}$$

where $H_{\text{MR}{\text{R}}_{1}}\left(f\right)=\left(t_{1}-\alpha {\text{e}}^{j\varphi }\right)/\left(1-\alpha t_{1}{\text{e}}^{j\varphi }\right)$ is the transmission of MRR_1_, *ϕ*_2_ is the phase difference between the two arms of MZI_2_, *t*_1_ is the equivalent self-coupling coefficient of MRR_1_, *φ* is the round-trip phase shift of the MRR_1_, and *α* is the round-trip amplitude transmission of MRR_1_. Here, *α* = exp(−*aL*/2), where *a* is the power attenuation coefficient and *L* is the perimeter of MRR_1_. Then the light is coupled with MRR_2_, and the output optical field *E*_5_ is expressed as

(4) $$\underline{E_{5}=H_{\text{MR}{\text{R}}_{2}}\left(f\right)E_{4}=\frac{\sqrt{2}}{4}\left(1+j{\text{e}}^{j\phi_{1}}\right)\frac{\left[\left(t_{1}+x{\text{e}}^{j\phi_{2}}\right)-\alpha \left(1+t_{1}{\text{e}}^{j\phi_{2}}x\right)\left.{\text{e}}^{j\varphi }\right)\right]\left(t_{2}-\alpha {\text{e}}^{j\varphi }\right)}{\left(1-\alpha t_{1}{\text{e}}^{j\varphi }\right)\left(1-\alpha t_{2}{\text{e}}^{j\varphi }\right)}E_{1},}$$

where $H_{\text{MR}{\text{R}}_{2}}\left(f\right)=\left(t_{2}-\alpha {\text{e}}^{j\varphi }\right)/\left(1-\alpha t_{2}{\text{e}}^{j\varphi }\right)$ is the transmission of MRR_2_, *t*_2_ is the equivalent self-coupling coefficient of MRR_2_. Notably, the parameters of MRR_2_ are designed to be the same as those of MRR_1_ except for the coupling coefficient (see in Supplementary Material Section 4). Therefore, the round-trip phase shift and round-trip amplitude transmission of MRR_2_ are equal to those of MRR_1_ and thus denoted by *φ* and *α*, respectively. For MRR_1_ and MRR_2_, assume that the cross-coupling coefficients of two directional couplers of the MZI coupler are both *k*_0_, which is represented by the yellow arrow in Figure 1(b). In the MZI couplers, the transmissions of both arms are assumed to be equal and denoted *α*_*b*_. By adjusting the electric power applied to the microheaters on the two arms of the MZI couplers, the coupling ratio can be changed from 0 to $2\alpha_{b}k_{0}\sqrt{1-{k_{0}}^{2}}$ (see in Supplementary Material Section 5) [21]. The cross section of the coupling region, which is marked by the red dashed curve in Figure 1(b), is shown in Figure 1(c). The waveguide width is designed as 500 nm to ensure single mode transmission, the etching depth is 150 nm, and the gap between the two bending waveguides is 350 nm. Finally, the optical signal is coupled out of the chip by GC_4_. A second-order APF can be obtained when the parameters of the device satisfy (see in Supplementary Material Section 6)

(5) $$\underline{\phi_{2}=k\pi ,}$$

(6) $$\underline{x={\left(-1\right)}^{k}\frac{\alpha^{2}t_{2}-t_{1}}{1-\alpha^{2}t_{1}t_{2}},}$$

(7) $$\underline{t_{2}=\alpha^{2}t_{1},}$$

where *k* is an arbitrary integer. Equation (7) indicates that MRR_2_ is overcoupled. Consequently, when Eqs. (5)–(7) are satisfied, the transmission of the second-order APF can be expressed as

(8) $$\underline{H_{2}\left(f\right)=\frac{E_{5}}{E_{1}}=\frac{\sqrt{2}\alpha^{2}\left(1-{t_{1}}^{2}\right)}{4\left(1-\alpha^{2}t_{1}t_{2}\right)}\left(1+j{\text{e}}^{j\phi_{1}}\right)\frac{\left(\alpha t_{1}-{\text{e}}^{j\varphi }\right)\left(\alpha t_{2}-{\text{e}}^{j\varphi }\right)}{\left(1-\alpha t_{1}{\text{e}}^{j\varphi }\right)\left(1-\alpha t_{2}{\text{e}}^{j\varphi }\right)},}$$

To make the principle understood intuitively, Figure 1(d) illustrates the principle of the second-order APF. When the optical beams in the upper and lower arms of MZI_2_ interfere with each other, the bandstop response induced by MRR_1_ can be converted to a bandpass response after MMI_5_. Then, the bandpass response is cascaded with MRR_2_. When the notch response of MRR_2_ is complementary to the bandpass response, an all-pass response is obtained. Additionally, the resonant wavelengths of MRR_1_ and MRR_2_ are aligned with each other to ensure that the amplitude response is constant. In the achieved APF, the phase variation in the FSR is twice that based on a single MRR [17, 18].

**Figure 2:**
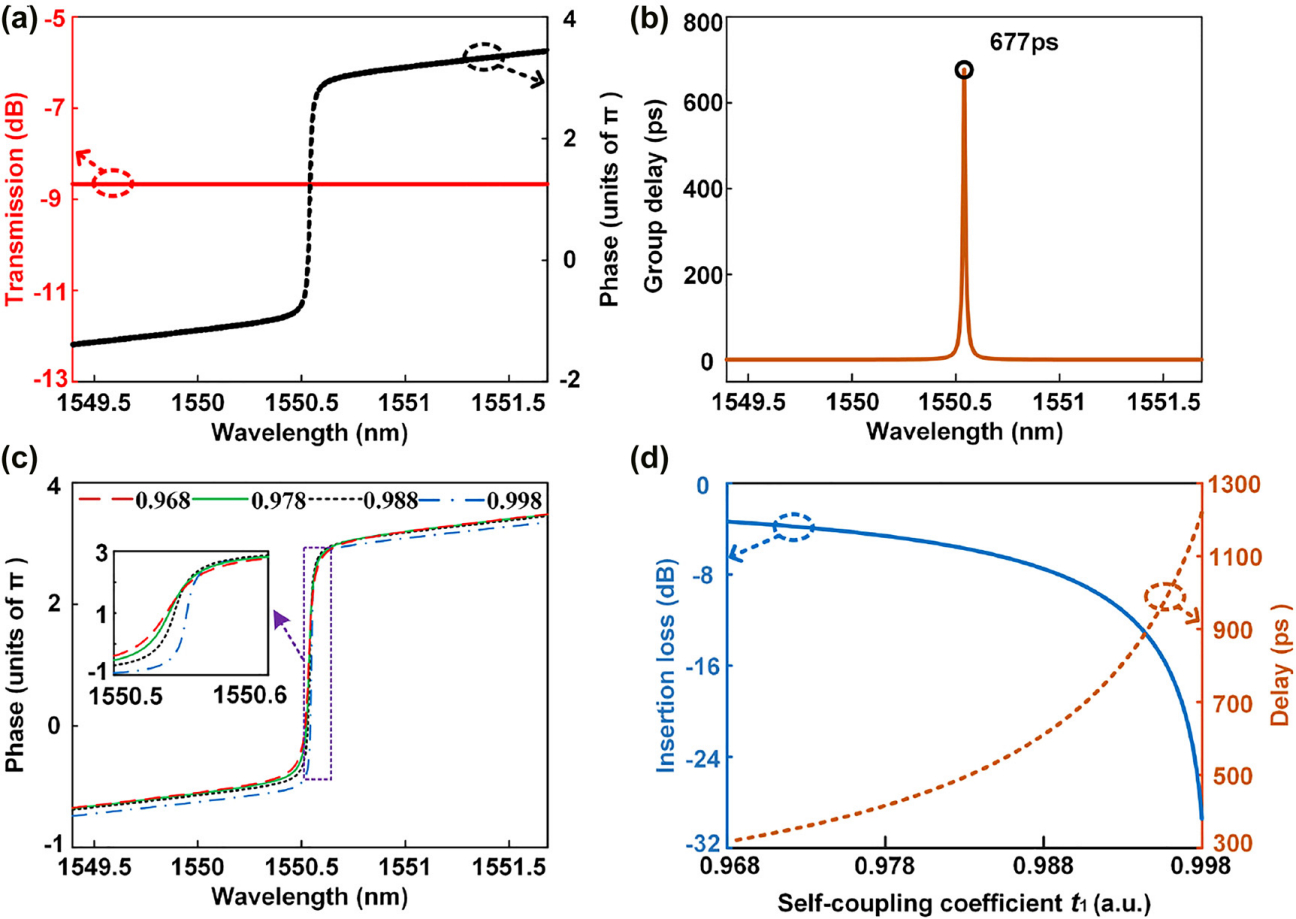
Simulation results of the second-order APF. (a) Amplitude (red solid curve) and phase (black short dashed curve) frequency responses when *t*_1_=0.99. (b) Corresponding group delay of the second-order APF. (c) Phase responses when *t*_1_ is set as 0.968 (red dashed curve), 0.978 (green solid curve), 0.988 (black short dashed curve) and 0.998 (blue dashed-dotted curve). (d) Variations in the insertion loss (blue solid curve) and delay (red short dashed curve) of the second-order APF when *t*_1_ is changed from 0.968 to 0.998.

To validate our analysis, we carry out simulations. Figure 2 shows the simulation results when *a* and *L* are set as 2.5 dB/cm and 251.32 μm, respectively. When *t*_1_ is set as 0.99, the amplitude and phase responses of the second-order APF are given by the red solid curve and the black short dashed curve in Figure 2(a), respectively. The amplitude response of the second-order APF is constant, and the phase shift reaches 4.83*π*, from 1549.395 to 1551.685 nm, which corresponds to the FSR of the MRRs. The results show that a second-order APF is obtained. The phase shift in the FSR is larger than 4*π*. This is caused by the MZI couplers for MRR_1_ and MRR_2_. Compared with a directional coupler, an MZI coupler has a complex coupling coefficient, whose phase can increase the phase shift range of the APF. The corresponding time delay is shown in Figure 2(b), and the peak is 677 ps. Notably, the phase response can be changed by adjusting *t*_1_. When *t*_1_ is set as 0.968, 0.978, 0.988 and 0.998, the phase responses of the second APF are given by the red dashed, green solid, black short dashed, and blue dashed-dotted curves in Figure 2(c), respectively. The inset is a zoomed-in view of the phase response from 1550.5 to 1550.6 nm. Figure 2(d) shows the variations in the insertion loss and the group delay of the second-order APF versus self-coupling coefficient *t*_1_. When *t*_1_ is adjusted from 0.968 to 0.998, the insertion loss (blue solid curve) and delay (orange short dashed curve) of the second-order APF increase from 3.3 to 29.4 dB and from 318 to 1219 ps, respectively.

In addition, the APF can be reconfigured to change the order. By adjusting the self-coupling coefficient of MRR_2_ to 0 or 1, which indicates that no resonance exists in MRR_2_, the APF can be reconfigured from second order to first order. Therefore, the flexibility of the APF is enhanced to adapt to different scenarios. Based on the theory in [18], a first-order APF can be realized when the parameters satisfy

(9) $$\underline{\phi_{2}=k\pi ,}$$

(10) $$\underline{x={\left(-1\right)}^{k}\frac{t_{1}\left(\alpha^{2}-1\right)}{1-\alpha^{2}{t_{1}}^{2}},}$$

Substituting Eqs. (9) and (10) into Eq. (4) and assuming *t*_2_ = 1, we can derive the transmission of the first-order APF, which can be expressed as

(11) $$\underline{H_{1}\left(f\right)=\;\frac{\sqrt{2}}{4}\left(1+j{\text{e}}^{j\phi_{1}}\right)\frac{\alpha \left(1-{t_{1}}^{2}\right)\left(\alpha t_{1}-{\text{e}}^{j\varphi }\right)}{\left(1-\alpha^{2}{t_{1}}^{2}\right)\left(1-\alpha t_{1}{\text{e}}^{j\varphi }\right)}.}$$

**Figure 3:**
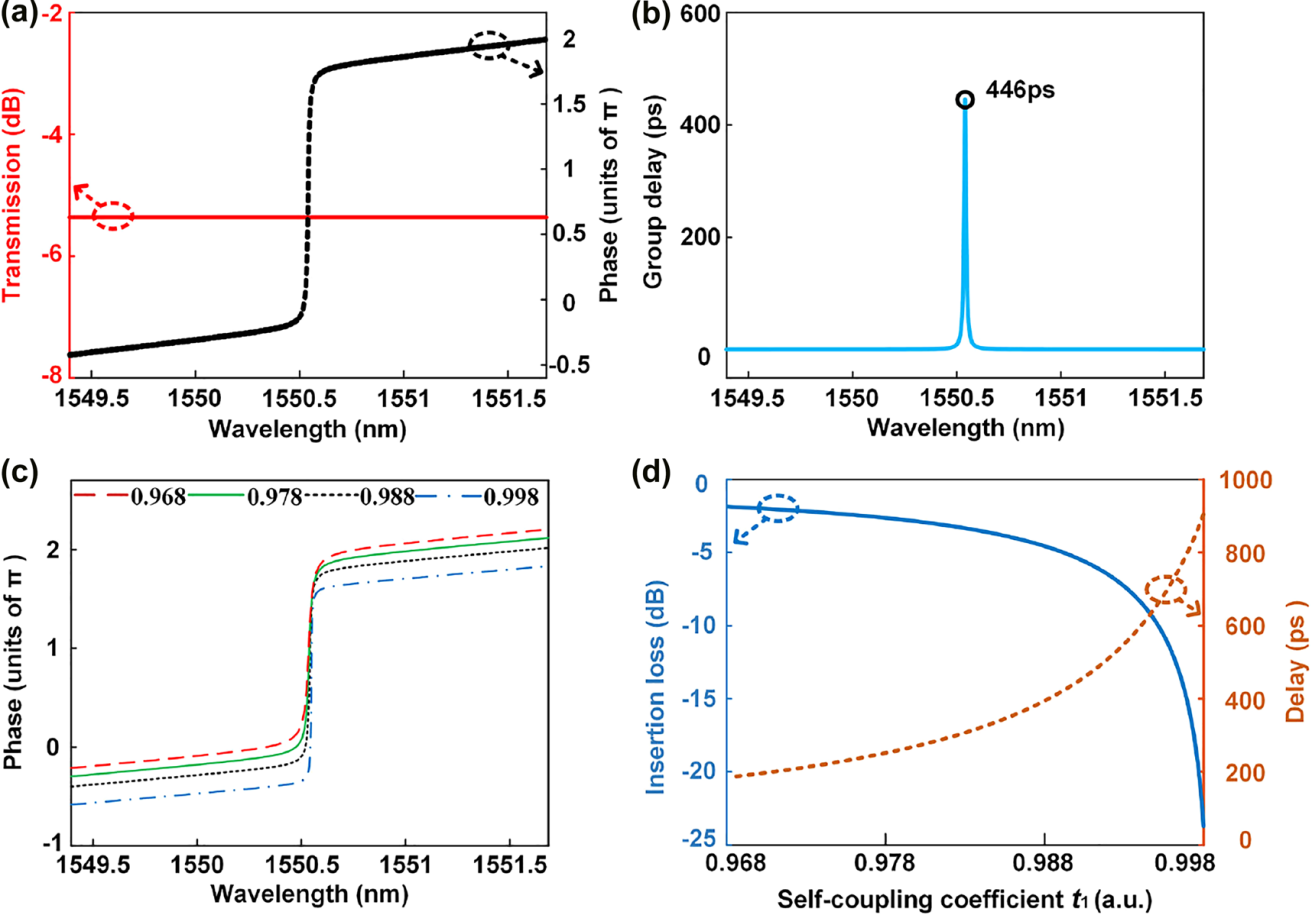
Simulation results of the first-order APF. (a) Amplitude (red solid curve) and phase (black short dashed curve) frequency responses when *t*_1_ = 0.99. (b) Corresponding group delay. (c) Phase responses when *t*_1_ is set as 0.968 (red dashed curve), 0.978 (green solid curve), 0.988 (black short dashed curve), and 0.998 (blue dashed-dotted curve). (d) Insertion loss (blue solid curve) and delay (red short dashed curve) of the first-order APF for different *t*_1_.

The simulation results of the first-order APF are shown in Figure 3. When the self-coupling coefficient of MRR_1_ is set as 0.99, which is the same as that in Figure 2(a), the amplitude and phase frequency responses are as shown in Figure 3(a). The amplitude response is constant and the phase shift, from 1549.395 to 1551.685 nm, is 2.42*π*. The phase shift is larger than 2*π*, which is also caused by the MZI coupler. The corresponding group delay is shown in Figure 3(b), and the peak is 446 ps. The phase response can also be adjusted by changing *t*_1_, which is the self-coupling coefficient of MRR_1_. When *t*_1_ is set as 0.968, 0.978, 0.988, and 0.998, which are the same as those in Figure 2(c), the phase responses of the first-order APF are given by the red dashed, green solid, black short dashed and blue dashed-dotted curves in Figure 3(c). Figure 3(d) shows the insertion loss and group delay of the first-order APF versus *t*_1_, and the trend is similar to that for the second-order APF. When the coupling coefficient of MRR_1_ remains the same, both the insertion loss and delay of the first-order APF are less than those of the corresponding second-order APF.

## 3 Results

**Figure 4:**
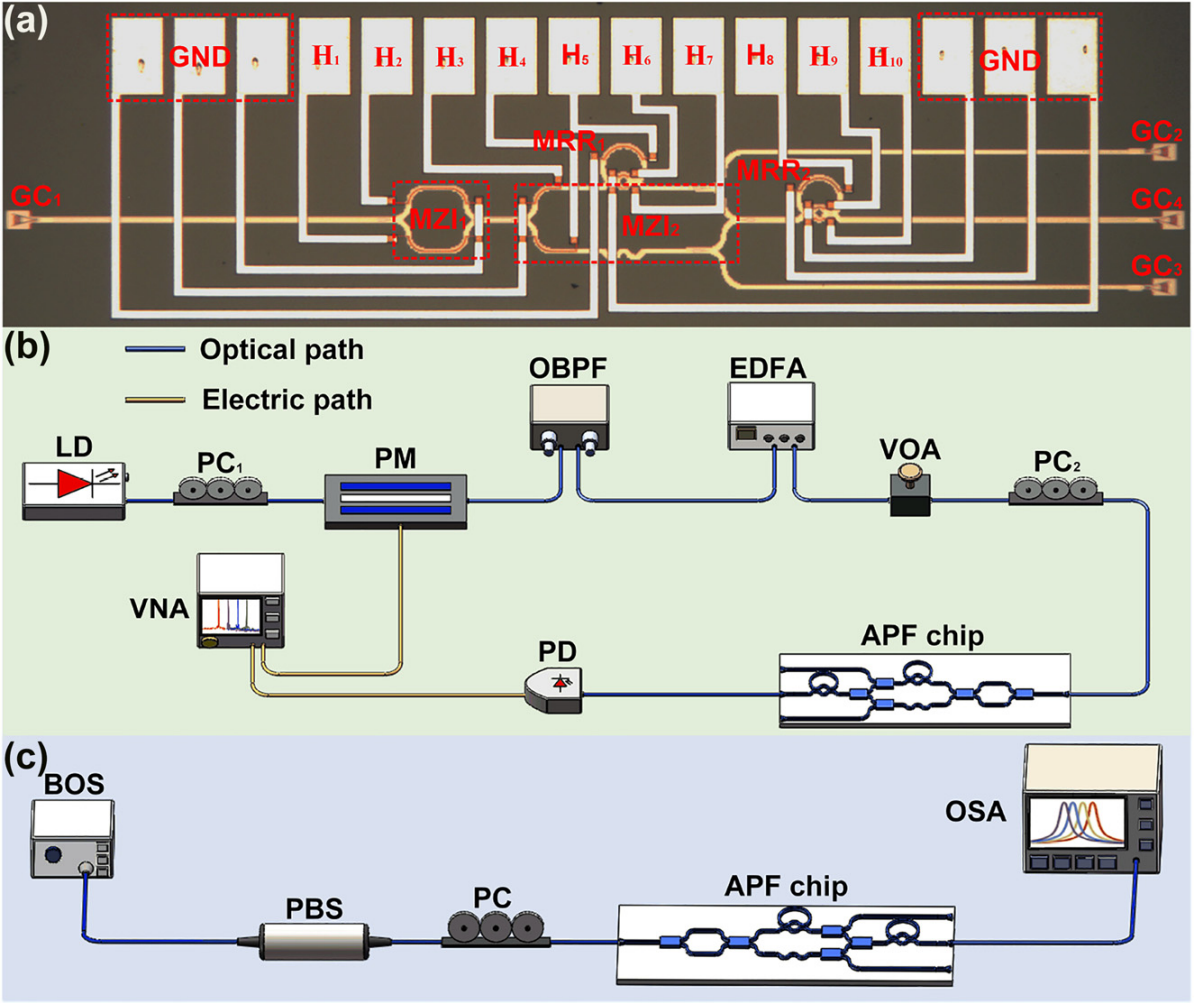
Fabricated device and measurements. (a) Optical micrograph of the fabricated device. (b) Experimental setup for measuring the frequency response and time delay of the fabricated device. (c) Experimental setup for measuring the optical transmission spectrum.

Based on the theoretical analysis, our proposed device is fabricated based on an SOI wafer. A micrograph of the fabricated device is shown in Figure 4(a). To measure the amplitude and frequency responses of the fabricated device, the experimental setup illustrated in Figure 4(b) is adopted. Continuous-wave (CW) light at 1550.00 nm emitted from a laser source (LS, Koheras BasiK E15) is launched into a phase modulator (PM, Covega Mach-40). A polarization controller (PC_1_) is used to align the state of polarization (SOP) of the CW light with the polarization axis of the PM. Then, an optical bandpass filter (OBPF, Alnair BVF-300CL) is used to eliminate the lower sideband of the phase-modulated signal, and a single-sideband (SSB) signal is obtained. An erbium-doped fibre amplifier (EDFA) and a variable optical attenuator (VOA) are used to adjust the optical power launched into the device. Then, the optical signal is coupled into the chip via GC_1_. After processing by the device, the output signal is coupled out of the chip via GC_4_ and launched into a photodetector (PD, SHF AG Berlin). Then, the converted electric signals are received and analysed by a vector network analyser (VNA, Anritsu, MS4647B). Figure 4(c) shows the experimental setup used to measure the optical transmission spectrum of the fabricated device. The light emitted from a broadband optical source (BOS) is polarized by a polarization beam splitter (PBS). Then, the SOP of the linearly polarized optical beam is adjusted to be aligned with the polarization axis of the optical waveguide by a PC. The broadband light is coupled into and out of the chip via GC_1_ and GC_4_, respectively. Finally, the optical transmission spectrum of the device is measured by an optical spectrum analyser (OSA, YOKOGAWA, AQ6370C).

**Figure 5:**
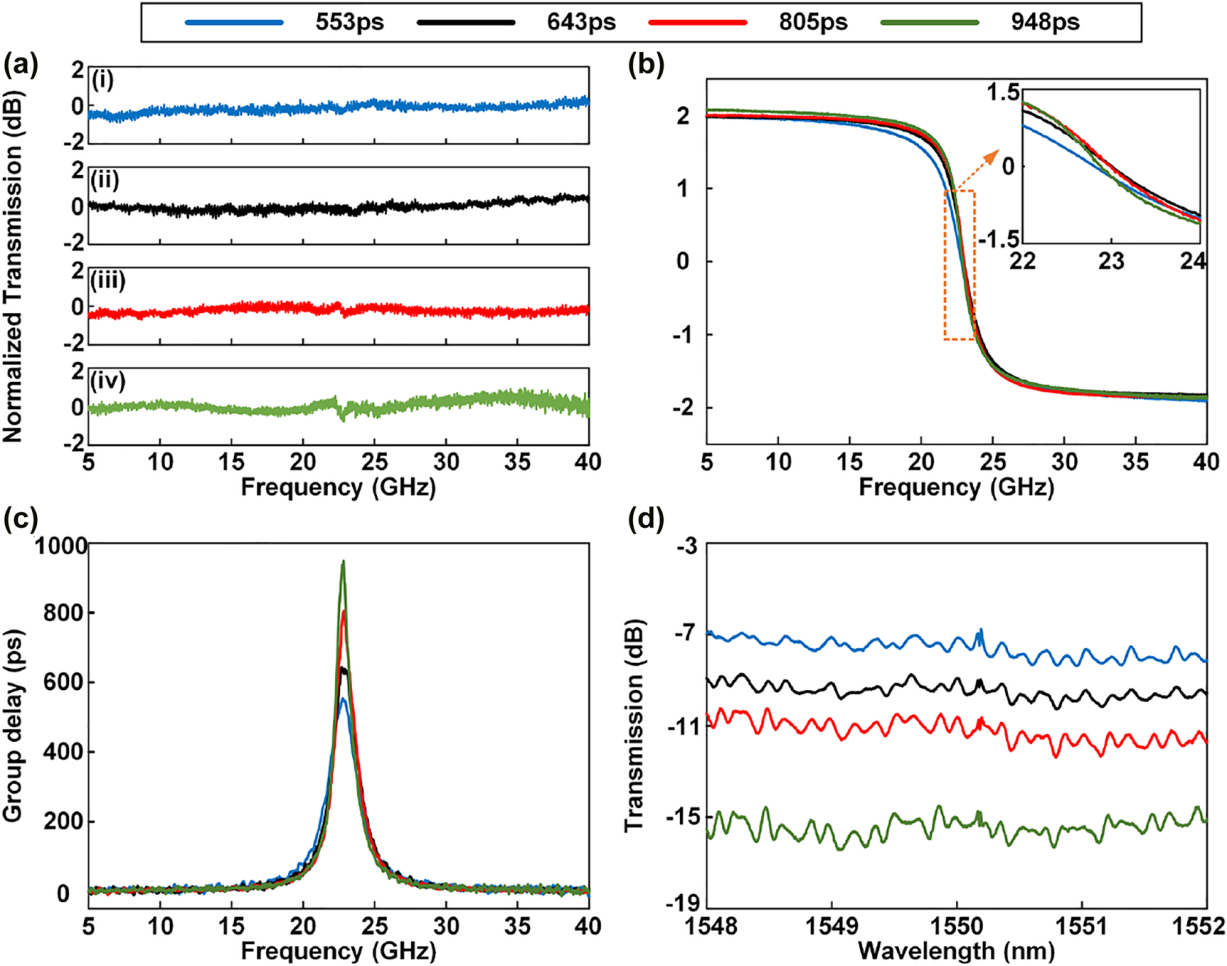
Experimental results of the second-order APF when the electric powers applied to H_2_, H_4_, H_5_, H_6_, H_7_, H_8_, and H_10_ are changed. (a) Amplitude frequency response. (b) Phase frequency response. (c) Group delay. (d) Optical transmission spectra measured by the OSA.

To realize a second-order APF, Eqs. (5)–(7) must be satisfied, which can be realized by adjusting the electric power applied to the microheaters on MZI_1_, MZI_2_, MRR_1_, and MRR_2_ (see in Supplementary Material Section 7). The measured results are shown in Figure 5. When the electric power applied to H_2_, H_4_, H_5_, H_7_, and H_8_ is 58.1, 63.3, 9.4, 23.4, and 28.9 mW, respectively, the achieved amplitude and phase responses are given by the blue solid curves in Figure 5(a) and (b), respectively. Obtainment of a second-order APF can be observed. The amplitude variation is within 1.4 dB, and the phase shift is 3.89*π*, from 5 to 40 GHz. A maximal time delay of 553 ps is achieved, shown by the blue solid curve in Figure 5(c). The corresponding insertion loss is 7.2 dB (see in Supplementary Material Sections 8 and 9), shown by the blue solid curve in Figure 5(d). By adjusting the electric power applied to H_2_, H_4_, H_5_, H_6_, H_7_, H_8_, and H_10_, the phase response and time delay are correspondingly changed (see in Supplementary Material Section 10). When the maximal time delay is adjusted to 643, 805, and 948 ps, shown by the black, red, and green solid curves in Figure 5(c), respectively, the corresponding amplitude responses, phase responses and transmission spectrum are given by the black, red, and green solid curves in Figure 5(a), (b) and (d), respectively. The inset of Figure 5(b) is a zoomed-in view of the phase response from 22 to 24 GHz. In Figure 5(a) and (d), we can observe amplitude variations in the measured results. This phenomenon is caused by the Fabry–Pérot (FP) cavity, environmental fluctuations and the amplitude variation around 22.75 GHz. The FP cavity results from the pair of input and output GCs. These amplitude variations cause fluctuations in the amplitude response of the APF and consequently induce signal power variation. In addition, the amplitude variation around 22.75 GHz shown in Figure 5(a) is mainly caused by the issue that the bandpass and bandstop responses are not totally complementary. This may be caused by misaligned resonant wavelengths of the two MRRs and insufficient adjustment resolution of the power supply to microheaters. In the second-order APF, MRR_2_ is overcoupled, and the bandpass response of MRR_1_ and the bandstop response of MRR_2_ are approximately complementary. Therefore, the extinction ratios of both the bandpass and the bandstop are enlarged as *t*_2_ increases. When the resonant wavelengths of MRR_1_ and MRR_2_ are misaligned, a high extinction ratio will lead to a large amplitude variation. Therefore, the measured magnitude of the amplitude variation in the phase shift region is enlarged as the group delay increases, as shown in Figure 5(a).

**Figure 6:**
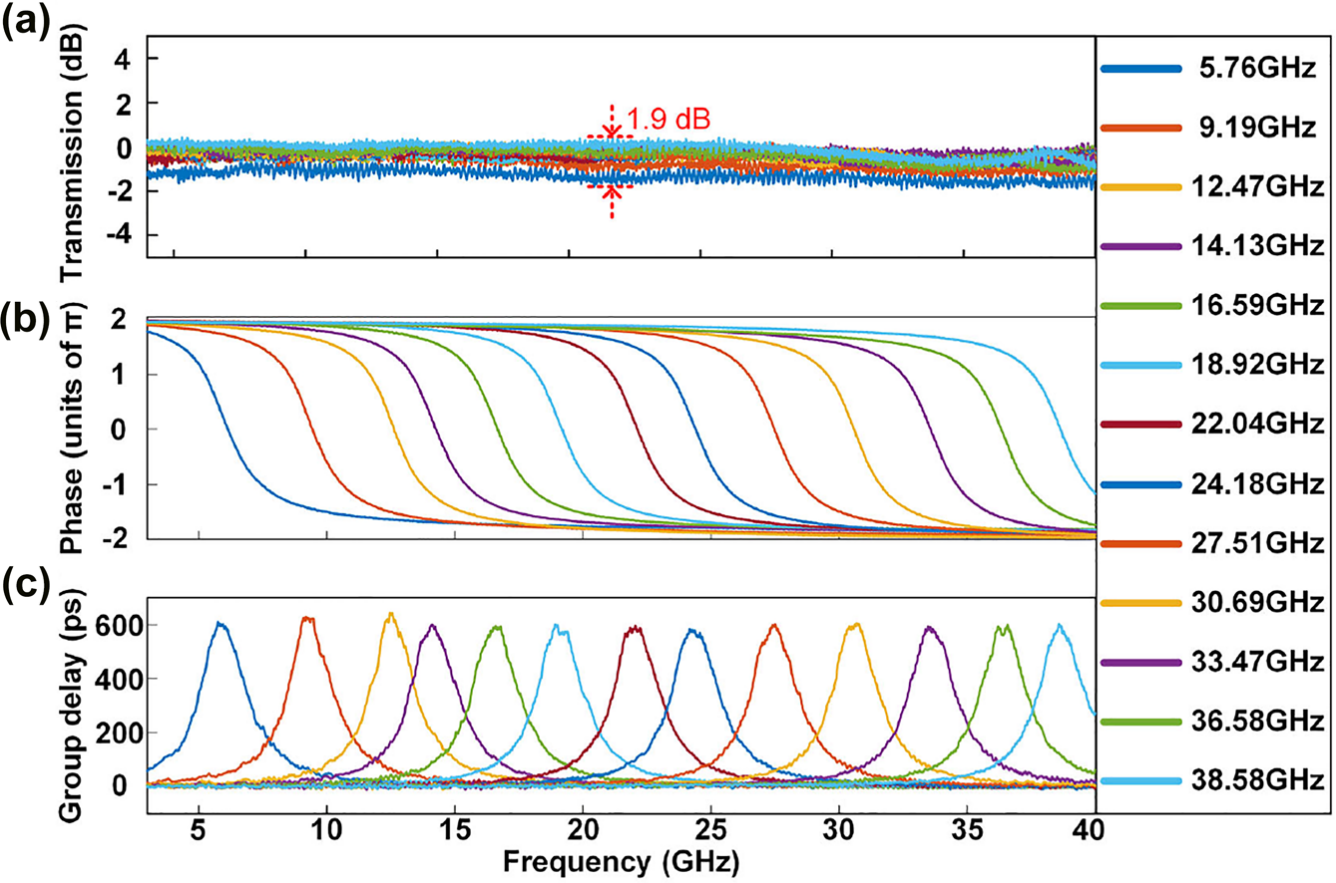
Experimental results of tuning the second-order APF from 5.7 to 38.6 GHz. (a) Amplitude frequency responses. (b) Phase frequency responses. (c) Group delay.

The phase response of the second-order APF can also be tuned. When the electric powers applied to microheaters H_1_, H_4_, H_5_, H_6_, H_7_, H_8_, and H_10_ are 42.9, 14.2, 58.9, 9.1, 56.6, 30.4, and 0 mW, respectively, the device works as a second-order APF, and the achieved time delay is 630 ps. Then, we adjust the electric powers applied to H_5_ and H_8_ to tune the resonant wavelengths of MRR_1_ and MRR_2_, respectively. To maintain all-pass transmission, both resonant wavelengths are adjusted to be aligned with each other. Based on the experimental setup shown in Figure 4(b), we adjust the centre of the phase shift area of the second-order APF from 5.7 to 38.6 GHz, as shown in Figure 6. Figure 6(a) shows that the transmission variation is less than 1.9 dB during the tuning process. The power variation is mainly caused by environmental fluctuations. For example, the mechanical vibration causes the SOP of the optical signal in fiber link varied. Thus, the transmitted power of the APF is changed because the fabricated GCs in the chip is polarization dependent. Figure 6(b) and (c) show the measured phase responses and time delay of the second-order APF when the electric powers applied to H_5_ and H_8_ are adjusted from 58.1 to 62.1 mW and from 25.6 to 31.2 mW, respectively.

**Figure 7:**
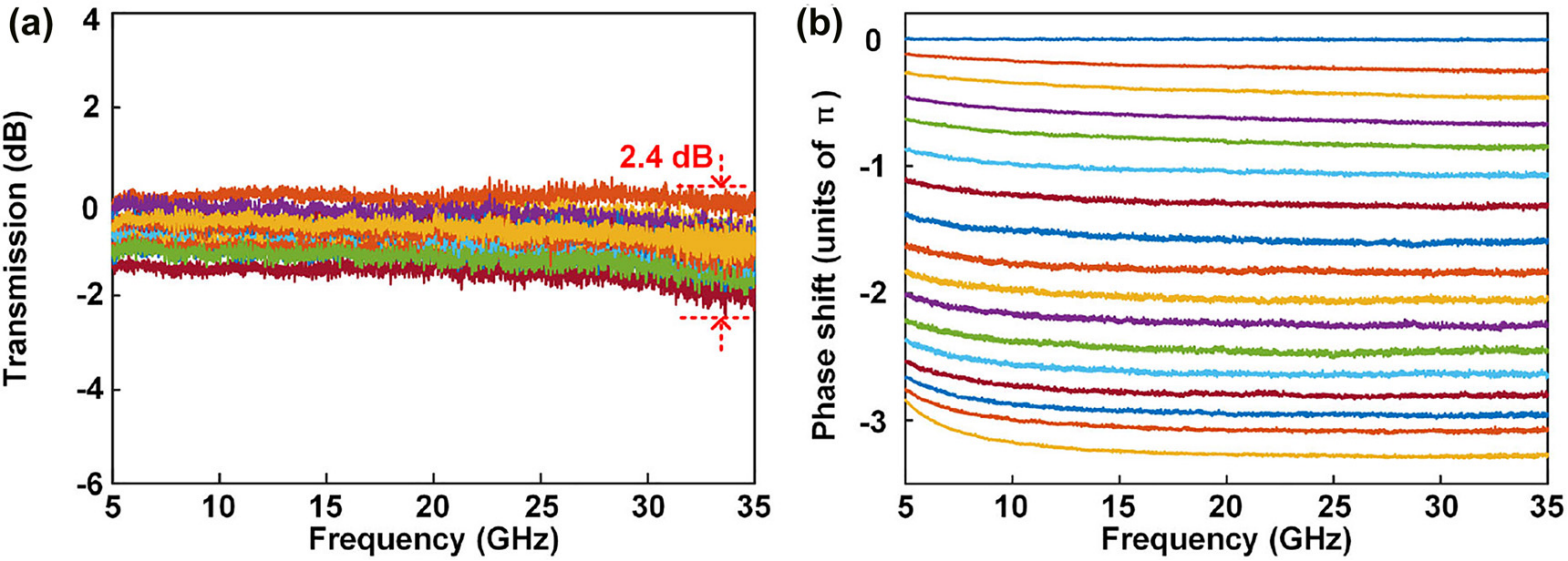
Measured frequency responses of the microwave photonic phase shifter. (a) Amplitude frequency responses. (b) Phase frequency responses.

The second-order APF is an ideal candidate to realize a broadband tuneable microwave photonic phase shifter. The optical carrier is placed within the phase shift area of the second-order APF, and the sideband is placed outside of the phase shift area [22]. By tuning the phase shift region of the second-order APF and adjusting the electric powers applied to H_5_ and H_8_, the phase difference between the optical carrier and the sideband is accordingly changed, and a tuneable microwave photonic phase shifter is obtained. Figure 7(a) and (b) show the amplitude and phase responses of the microwave photonic phase shifter. The microwave phase can be shifted from 0 to 3.27*π*, while the RF power variation is 2.4 dB, which is larger than the predicted result. The RF power variation is mainly caused by three factors. The first is environmental fluctuations. The second is the relative wavelength drift between the resonant wavelengths of MRR_1_ and MRR_2_. Additionally, the FP effect caused by reflections of the GC pair also contributes to the power variation. Notably, in the previously reported microwave photonic phase shifter based on the first-order APF [17, 18], the microwave phase shift is less than 2*π*. In contrast, the microwave photonic phase shifter based on the second-order APF has a much larger phase shift range, and the phase shift exceeds 2*π*, which is significantly important for phased array antennas.

**Figure 8:**
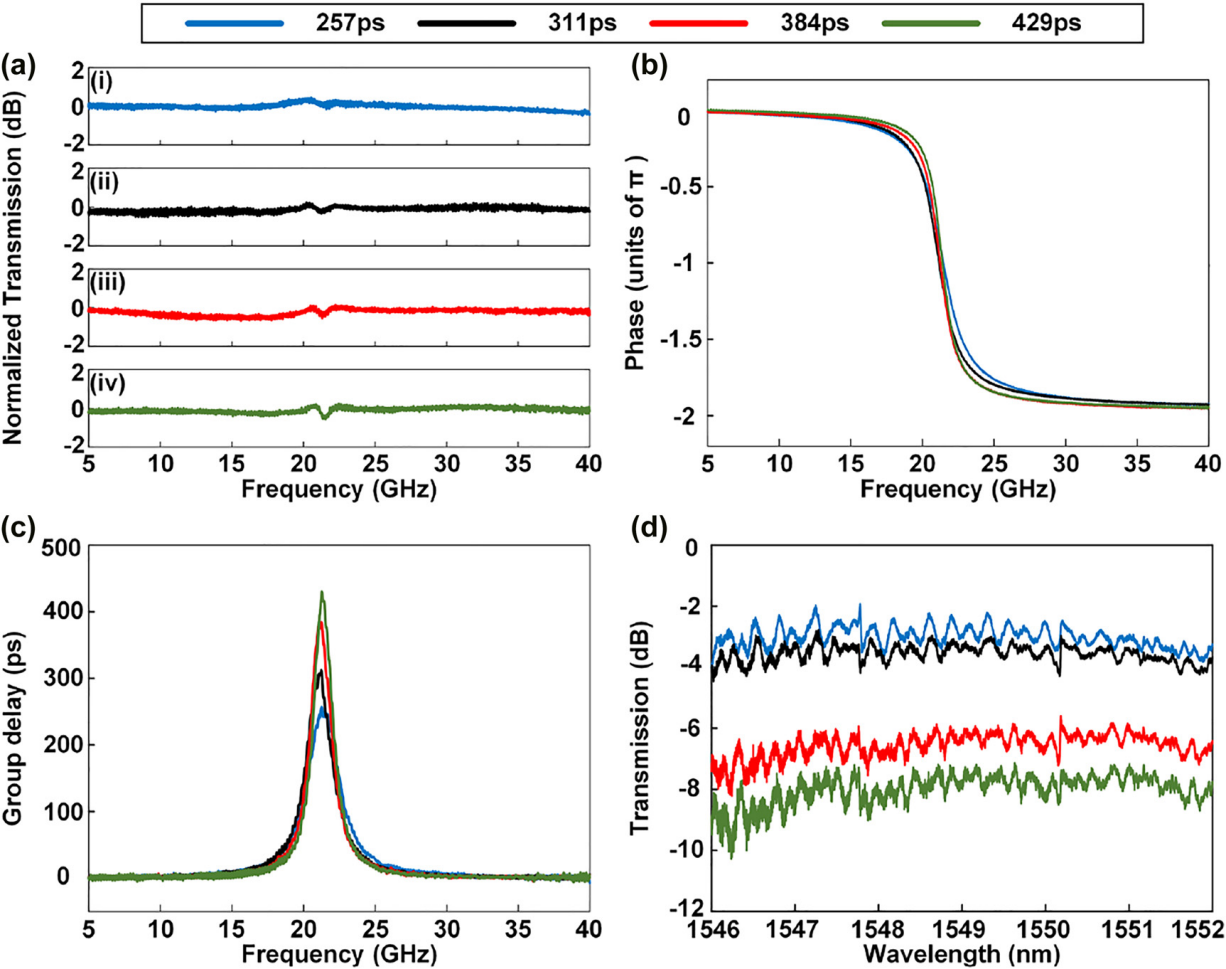
Experimental results of the first-order APF when the electric powers applied to H_2_, H_4_, H_5_, and H_6_ are changed. (a) Amplitude frequency response. (b) Phase frequency response. (c) Group delay. (d) Optical transmission spectra measured by the OSA.

Additionally, the second-order APF can be reconfigured to a first-order APF, which significantly enhances the flexibility of the APF. The first-order APF can be obtained when the self-coupling coefficient between the waveguide and MRR_2_ is 0 or 1. In the experiment, when the electric power applied to H_10_ is 25.1 mW, the self-coupling coefficient between the waveguide and MRR_2_ is almost 1. Therefore, the optical signal is not resonant in MRR_2_. When the electric powers applied to microheaters H_2_, H_4_, and H_5_ are adjusted to 58.8, 68.3, and 26.6 mW, respectively, a first-order APF with a 257 ps delay can be realized, as shown by the blue solid curve in Figure 8. By adjusting the electric powers applied to H_2_, H_4_, H_5_, and H_6_ (see in Supplementary Material Section 10), the phase response of the first-order APF can be changed, and the corresponding time delay is correspondingly adjusted. When the electric powers applied to H_2_, H_4_, and H_6_ are adjusted from 58.8 to 62.5 mW, 68.3–76.0 mW, and 0–12.4 mW, respectively, the amplitude and phase responses are as shown in Figure 8(a) and (b), respectively. Figure 8(a) shows that the maximum amplitude variation is 0.9 dB during the adjustment process. Two factors contribute to the amplitude variation occurred at 22 GHz. The first is the notch existing in the transmission spectrum of MRR_2_ because the cross-coupling coefficient between the waveguide and MRR_2_ cannot be exactly adjusted to 0. Therefore, slight optical signal is coupled into MRR_2_ and a transmission notch is generated. The second factor is the limited resolution of adjusting the power applied to the microheaters, which caused the corresponding parameters slightly deviate from the desired values. Figure 8(b) shows that the roll-off rate of the phase response is consequently changed. As a result, the corresponding time delay is adjusted from 247 to 429 ps, as shown in Figure 8(c). Figure 8(d) shows the corresponding optical transmission spectrum of the first-order APF measured by the OSA. The FP effect is also observed in the transmission spectrum. The insertion loss also increases as the time delay increases. Notably, if we simply cascade two first-order APFs which contains the part from GC_1_ to MMI_5_, the device will occupy much more size and more heaters will be required than those of the structure displayed in Figure 1(a). Therefore, our proposed device shows advantages of more compact size, simpler structure, and less power consumption.

## 4 Conclusions

In conclusion, we have realized a tuneable and reconfigurable optical second-order APF based on SOI. Both simulations and experiments are carried out to demonstrate the APF. Compared with the second-order APF achieved by simply cascading two first-order APFs, our proposed second-order APF is much simpler and more compact. The second-order APF-based adjustable delay line and microwave photonic phase shifter are also investigated. The results show that when the time delay is adjusted from 553 to 948 ps, the amplitude variation is within 1.7 dB. Based on the second-order APF, a microwave photonic phase shifter with a phase shift from 0 to 3.27*π* is achieved, and the corresponding RF power variation is less than 2.4 dB. The second-order APF can also be reconfigured to a first-order APF when the self-coupling coefficient of MRR_2_ is set as 0 or 1. In the first-order APF, the amplitude variation is less than 0.9 dB, and the group delay can be adjusted from 247 to 429 ps. Another advantage is that the proposed approach can be easily extended to realize APFs with even higher orders. Compared with high-order APFs obtained by simply cascading first-order APFs, our proposed approach is much simpler and more compact since fewer optical elements are used.

## Supplementary Material

Supplementary Material Details
