# Supplementary material for: Reconfigurable second-order optical all-pass filter
Source: Nanophotonics. 2022 May 27;11(13):3115–25. doi: 10.1515/nanoph-2022-0140 (PMC11501854; doi:10.1515/nanoph-2022-0140)
Supplement: Supplementary file 1 — Supplementary Material Details [file j_nanoph-2022-0140_suppl.docx]

Supplementary Material

1. EXTENSION OF OUR PROPOSED DEVICE TO HIGH ORDER APFS


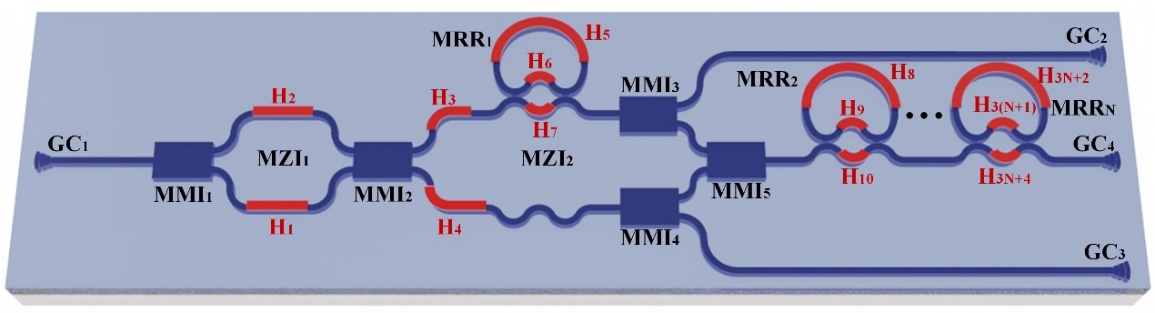


Fig. S1 Schematic diagram of higher order APF.

Fig. S1 displays the schematic diagram of the higher order APF based on our proposed approach. It consists of two MZIs and N MRRs with equal ring length. Each MRR is designed with a MZI coupler. Based on Eq. (4), the transmission of a Nth-order APF can be obtained

where *t*_1_, *t*_2_, *t*_3_, …, and *t*_N_ are coupling coefficients of MRR_1_,MRR_2_,MRR_3_,…, and MRR*_N_*, *x* is the power splitting ratio of two arms of MZI_2_, *ϕ*_1_ and *ϕ*_2_ are the phase difference between the two arms of MZI_1_ and between the two arms of MZI_2_, *φ* is the round-trip phase shift of the all the MRRs, and *α* is the round-trip amplitude transmission. The Z transform of Eq. (S1) can be expressed as

where. From the Eq. (S2), the zeros and poles of the device can be obtained

$$\left\{\begin{matrix} p_{1}=\frac{1}{\alpha t_{1}},\text{ (S4.1)} \\ p_{2}=\frac{1}{\alpha t_{2}},\text{ (S4.2)} \\ p_{3}=\frac{1}{\alpha t_{3}},\text{ (S4.3)} \\ \begin{matrix} \cdot\text{ } \\ \cdot\text{ } \\ \cdot\text{ } \\ p_{N}=\frac{1}{\alpha t_{N}},\text{ (S4.N)} \end{matrix} \end{matrix}\right.$$

Based on Eqs. (S3.1)-(S3.N) and (S4.1)-(S4.N), we can see that the location of the zero of MRR_1_ can be changed arbitrarily by tuning the electrode H_1_ (or H_2_) on MZI_1_ and the electrode H_3_ (or H_4_) on MZI_2_. The working principle of the Nth-order optical APFs is: the pole of MRR_1_ and the zero of MRR_2_ satisfy the APF condition, the pole of MRR_2_ and the zero of MRR_3_ satisfy the APF condition, and so on, until the pole of MRR_N-1_ and the zero of the MRR_N_ satisfy the APF condition. Finally, in order to make the zero of MRR_1_ and the pole of the MRR_N_ match the APF condition, we can adjust the electric power on H_1_ (or H_2_) and H_3_ (or H_4_) to change the zero position of MRR_1_, so as to realize a Nth-order APF. Therefore, we can obtain the equations as follows

$$\left\{\begin{matrix} z_{2}=\frac{1}{p_{1}^{*}},\text{ (S5.1)} \\ z_{3}=\frac{1}{p_{4}^{*}},\text{ (S5.2)} \\ z_{4}=\frac{1}{p_{3}^{*}},\text{ (S5.3)} \\ \begin{matrix} \cdot\text{ } \\ \cdot\text{ } \\ \cdot\text{ } \\ \begin{matrix} z_{N}=\frac{1}{p_{N-1}^{*}},\text{ (S5.N-1)} \\ z_{1}=\frac{1}{p_{N}^{*}},\text{ (S5.N)} \end{matrix} \end{matrix} \end{matrix}\right.$$

Combining Eqs. (S3.1)-(S3.N), (S4.1)-(S4.N) and (S5.1)-(S5.N), we can obtain a Nth-order APF when the parameters satisfy

where *k* is an arbitrary integer. When Eqs. (S6.1)-(S6.N) are satisfied, we can obtain the transmission of the Nth-order APF

In addition, the order of the high-order APF can also be reconfigured. By tuning the coupling coefficient of the cascading MRRs to 0 or 1, the high-order APF will be reconfigured to a lower order APF, which will undoubtedly make the device more flexible in applications.

1. **DEVICE FABRICATION**

The device was commercially fabricated at Chongqing United Microelectronics Center (CUMEC). A standard SOI wafer with a 220-nm-thick silicon layer and a 2-μm-thick buried oxide layer was used. All the silicon waveguides were rib waveguides, and the width and etching depth of the waveguide were 500 nm and 150 nm, respectively. Then, SiO_2_ with a thickness of 1 µm was deposited as the upper cladding layer of the waveguides. To tune the device, a heater made of TiN was deposited on the upper SiO_2_ layer. Then, the related metal routing, which provides the current transfer path, was made of AlCu. Finally, another SiO_2_ layer was deposited on the metal layer to prevent the metal from oxidizing.

1. **INFLUENCES OF FABRICATION ERROR**

As is known, the fabrication error is inevitable when the SOI chip is fabricated. In terms of MMI, the fabrication error can cause the coupling value of MMI deviated from the targeted value. To reduce this deviation, we designed MZI_1_ and MZI_2_ to adjust the power splitting ratio between the two arms of MZI_2_. By adjusting the electric power applied to H_1_ or H_2_, the power splitting ratio can also be adjusted to the target value of the APF. Secondly, the fabrication error can also induce the optical waveguides with different widths in the two arms of MZI_2_, which induces an optical phase difference between the two arms of MZI_2_. To eliminate the fabrication error induced phase difference, we deposited a microheater on the two arms of MZI_2_ to make the phase difference is 0 or π. The fabrication error can also induce the coupling coefficient between the MRR and bus waveguide deviated from the targeted value. To make the coupling coefficient between MRR_1_ and bus waveguide, and the coupling coefficient between MRR_2_ and bus waveguide are equal to their targeted values, MZI couplers are used. Additionally, the fabrication error can cause the misaligned resonant wavelengths of two MRRs. To maintain all-pass transmission, we deposited microheaters on two MRRs to align both resonant wavelengths with each other. Therefore, the fabrication variation caused coupling coefficient, phase deviations and wavelength misalignment can be eliminated in our device.

1. **IMPACT OF UNEQUAL TRANSMISSION LOSSES OF THE TWO MRRS**

In this section, we explore the impact of unequal round-trip transmission coefficients of the two MRRs. The transfer functions of the two MRRs are expressed as

where *α*_1_ and *α*_2_ are the round-trip transmission coefficients of MRR_1_ and MRR_2_, respectively. And the all-pass conditions can be modified as

According to the theoretical analysis, a second-order APF can be obtained based on the device with unequal round-trip transmission coefficients of the two MRRs. Thus, we can omit the impact of unequal transmission losses of the two MRRs and assume that the transmission losses of the two MRRs are equal.

1. COUPLING COEFFICIENT OF THE MZI COUPLER

As shown in Fig. S2, the self- and cross-coupling coefficients of the MZI coupler are *t*_0_ and *k*_0_, which are represented by the red and yellow arrows, respectively. The length and transmission coefficients of the two arms of the balanced MZI coupler are denoted *L_b_* and *α_b_*, respectively. Based on the transfer matrix, we can derive the equivalent coupling matrix of the MZI coupler, which can be expressed as

where *ϕ_r_* and *ϕ_b_* are the phase variations induced by the upper and lower arms of the MZI coupler, respectively. *A*, *B*, *C* and *D* represent the transmission from port 1 to port 2, port 1 to port 4, port 3 to port 2 and port 3 to port 4, respectively. Then, Eq. (S11) can be simplified as

The self- and cross-coupling coefficients of the MZI coupler are both complex values. From Eq. (S12), we can derive the effective amplitude coupling from port 1 to port 4 as. By changing the phase difference between the two arms of the MZI, the cross-coupling ratio can be changed from 0 to$2\alpha_{b}t_{0}k_{0}$.





Fig. S2. Schematic diagram of the MZI coupler.

1. DEMONSTRATION OF THE SECOND-ORDER APF

The Z transform of an APF can be expressed as the ratio of two polynomials [1],

$$H(z)=e^{j\zeta}\frac{d_{N}^{*}+d_{N-1}^{*}z^{-1}+\cdot\cdot\cdot+z^{-N}}{1+d_{1}z^{-1}+\cdot\cdot\cdot+d_{N}z^{-N}}=e^{j\zeta}\frac{\prod_{k=1}^{N} (-z_{k}^{*}+z^{-1})}{\prod_{k=1}^{N} (1-z_{k}^{*}z^{-1})},\text{ (S13)}$$

where $z_{k}^{*}$ and $\frac{1}{z_{k}^{*}}$ are the zeros and poles of the APF. From Eq. (S13), we can see that the zero locations of the APFs are mirror images about the unit circle from the pole locations. The transmission of an all-pass MRR can be expressed by [2]

where *t* is the self-coupling coefficient, *φ* is the round-trip phase shift of the MRR and *α* is the round-trip amplitude transmission. The Z transform of Eq. (S14) can be expressed as

$$H_{MRR}(z)=\frac{t-\alpha z}{1-\alpha tz}\text{, (S15)}$$

In the z domain, . The pole and zero of the all-pass MRR can be expressed as$p=\frac{1}{\alpha t}$and$z=\frac{t}{\alpha}$, respectively. If *α* is 1, then the APF condition$p=\frac{1}{z^{*}}$is certainly satisfied [1]. However, this all-pass state cannot be achieved in practice because there is always loss in optical waveguides. The use of interference is demonstrated to be an effective solution to tune the zero location without changing the pole location [3]. Thus, by using two cascaded tuneable MZIs, the zero location can be arbitrarily adjusted.

In the designed device shown in Fig. 4(a), MMI_3_ and MMI_4_ are used to monitor the transmission of the two arms of MZI_2_ and can be eliminated in practice. Therefore, we omit the optical loss caused by MMI_3_ and MMI_4_. The optical fields coupled into the chip by GC_1_ and out of the chip by GC_4_ are denoted *E_in_* and *E_out_*, respectively. The frequency response of the proposed device can be expressed as

where *ϕ*_1_ and *ϕ*_2_ are the phase differences between the two arms of MZI_1_ and MZI_2_, respectively, and can be tuned by applying voltage to microheaters H_1_ (or H_2_) and H_3_ (or H_4_). The ratio of the optical fields in the lower and upper arms of MZI_2_ is expressed as

Substituting Eqs. (S8), (S9) and (S17) into Eq. (S16), we can derive

where *t*_1_ and *t*_2_ are the equivalent self-coupling coefficients of MRR_1_ and MRR_2_, respectively. The Z transform of Eq. (S18) can be expressed as

From Eq. (S19), we can obtain the zeros and poles of the device,

As mentioned above, the zero and pole of an all-pass MRR cannot satisfy the requirement of an APF unless *α*=1. Thus, we can obtain

$$\left\{\begin{matrix} z_{1}=\frac{1}{p_{2}^{*}},\text{ (S21.1)} \\ z_{2}=\frac{1}{p_{1}^{*}},\text{ (S21.2)} \end{matrix}\right.$$

Combining Eqs. (S20) and (S21), we can obtain Eqs. (5)-(7). Therefore, when Eqs. (5)-(7) are satisfied, a second-order APF is realized.

Notably, the obtained all-pass condition is concise and intuitive. To obtain the impact of the microheaters on the second-order APF, the transmission of the APF shown in Fig. 4(a) is further derived. Substituting Eq. (S12) into Eqs. (S8) and (S9), we can derive the transfer functions of MRR_1_ and MRR_2_, which can be expressed as

where *ϕ_a_* is the phase in the upper arm of the MZI coupler for MRR_1_, *α_r_* is the and Δ*ϕ*_0_, Δ*ϕ*_1_ and Δ*ϕ*_2_ are the phase differences between the two upper arms of the MZI couplers for MRR_1_ and MRR_2_, between the upper and lower arms of the MZI coupler for MRR_1_ and between the upper and lower arms of the MZI coupler for MRR_2_, respectively. Substituting Eqs. (S22), (S23) and (S17) into Eq. (S16), we can derive

Performing Z transformation on Eq. (S24), the derived poles and zeros can be expressed as

Combining Eqs. (S21) and (S25), the all-pass condition of the proposed second-order APF based on MZI couplers can be derived and expressed as

Notably, adjusting Δ*ϕ*_1_ can change the coupling coefficient between the bus waveguide and MRR_1_. If Eqs. (S26.1)-(S26.3) are still satisfied when adjusting Δ*ϕ*_1_, then the phase response of the APF can be adjusted. Based on Eqs. (S24) and (S26), the simulation results are displayed in Fig. 2.

1. ADJUSTING THE DEVICE TO REALIZE A SECOND-ORDER APF

To achieve a second-order APF, we adjust the electric power applied to H_10_ to make MRR_2_ over coupled at first. When MRR_2_ is over coupled, the phase response has a phase shift of almost 2π. Secondly, the phase difference between the two arms of MZI_2_ should be adjusted to 0 or π. When we adjust the electric power applied to H_4_, the shape of the transmission spectrum of the combined MRR_1_ and the lower arm of MZI_2_ is changed. Notably, to observe the shape of transmission spectrum, the resonant wavelength of MRR_2_ is adjusted to be far away from the resonant wavelength of MRR_1_. When the filter shape of the interfered optical spectrum measured at GC_4_ (bandpass or bandstop) is symmetrical about resonant wavelength, the phase difference between the two arms of MZI_2_ is 0 or π. Thirdly, the power splitting ratio between the two arms of MZI_2_ should be adjusted. By adjusting the electric power applied to H_1_, the splitting ratio *x* is adjusted until the extinction ratio of the bandpass response is the same as the rejection ratio of the bandstop response of MRR_2_. If the -3dB bandwidths of the bandpass and the bandstop responses are unequal, we need to adjust the electric power on H_6_ or H_7_ to adjust the coupling coefficient of MRR_1_. To make the rejection ratios of the bandpass and the bandstop responses equal to each other and *φ*_2_ equal to 0 or π respectively, adjust the electric power on H_1_ and H_4_ to compensate the optical amplitude and phase variations caused by the changed electric power applied to H_6_ or H_7_. Finally, when we obtain a suitable value of *t*_1_ to make the -3dB bandwidths of the bandpass and the bandstop responses equal, adjust the electric power applied to H_5_ and H_8_ to make the resonant wavelengths of MRR_1_ and MRR_2_ aligned with each other. Then, a second-order APF is obtained. However, the existed thermal crosstalk makes the transmission of the APF not constant. To compensate the thermal crosstalk and optimize the response of the second-order APF, we still need to finely adjust the electric power on H_1_, H_4_, H_5_, H_6_, H_7_, H_8_ and H_10_ until the transmission is flattened. To reduce the influence of thermal crosstalk, several methods can be used, such as etching air trenches [4], and increasing the spacing between the heaters in device fabrication.

1. MEASURING THE OPTICAL TRANSMISSION SPECTRUM OF THE APF

The optical transmission spectrum, which is used to reflect the insertion loss of the proposed device, was obtained by subtracting the transmission spectrum of the reference waveguide from the measured APF transmission spectrum. Due to the influence of the fabrication error on the MMIs, the optical signal cannot be completely sent into the lower arm of MZI_2_. Thus, the self-coupling coefficients of MRR_1_ and MRR_2_ were tuned to close to 1 and the electric power on H_1_ (or H_2_) was adjusted to maximize the output power from GC_4_. Then, the proposed device shown in Fig. 4(a) was equivalent to a reference waveguide from GC_1_ to GC_4_, and the measured optical transmission spectrum of the reference waveguide is displayed in Fig. S3.





Fig. S3. Measured transmission spectrum of the reference waveguide.

1. THE INSERTION LOSS OF THE PROPOSED APF

Fig. S4 Simulation results. When the self-coupling coefficient of MRR_1_ is set as 0.99, the variations of the insertion loss (blue solid curve) and the delay (orange short dashed curve) of the second-order APF (a) and the first-order APF (b) versus power attenuation.

Directly, the insertion loss of the device is caused by three factors. The first is the attenuation of the notch of MRR_1_. The second is the optical inference between the optical signals in the two arms of MZI_2_. The third is the attenuation of the notch of MRR_2_. Fig. 2a and Fig. 3a show the simulated amplitude and phase responses of the second and the first order APFs, respectively. Compared with the second-order APF, the splitting ratio *x* of the first-order APF is decreased, and the insertion loss introduced by the inference between the optical signals in the two arms of MZI_2_ is reduced correspondingly. Additionally, in the first-order APF, the self-coupling coefficient of MRR_2_ is adjusted to 1, and the loss caused by MRR_2_ is quite small and can be omitted. Therefore, the first-order APF has lower insertion loss than the second-order APF. Adjusting the coupling coefficient to change the time delay will change the power attenuation of the notch of MRRs, so the insertion loss of APFs will be changed as the delay.

Essentially, the loss of optical waveguide is the ultimate limiting factor of the insertion loss of the APF, which can be demonstrated by simulation results shown in Fig. S4. When *t*_1_ is set as 0.99, Figs. S4(a) and (b) show the insertion loss (blue solid curve) and delay (orange short dashed curve) of the second and the first order APF for different power attenuation coefficient *a*, respectively. When *a* is adjusted from 0 to 5, the insertion loss of the second-order APF is increased from 0 to 12.9 dB and the delay is reduced from 1647 to 441 ps, respectively. Meanwhile, the insertion loss of the first-order APF is increased from 0 to 8.7 dB and the delay is reduced from 824 to 306 ps, respectively. Therefore, waveguides with lower loss can effectively improve the tradeoff between loss and group delay. If other material with lower loss, such as silicon nitride (Si_3_N_4_), is used to fabricate the device, the tradeoff between loss and group delay will be certainly improved.

1. ADJUSTING THE SECOND- AND FIRST-ORDER APFS

Table S1. Electric power applied to the microheaters and power variation of the second-order APF

| H_1_  (mW) | H_4_  (mW) | H_5_  (mW) | H_6_  (mW) | H_7_  (mW) | H_8_  (mW) | H_10_  (mW) | Delay (ps) | Power variation (dB) | Insertion loss (dB) |
| --- | --- | --- | --- | --- | --- | --- | --- | --- | --- |
| 58.1 | 63.3 | 9.4 | 0 | 23.4 | 28.9 | 0 | 553 | 1.4 | 7.2 |
| 61.2 | 67.0 | 8.6 | 0 | 26.8 | 27.9 | 6.8 | 643 | 1.3 | 8.9 |
| 40.8 | 11.9 | 55.8 | 9.8 | 54.7 | 26.8 | 15.2 | 805 | 1.0 | 10.7 |
| 40.3 | 11.7 | 53.2 | 9.8 | 53.8 | 24.3 | 18.0 | 948 | 1.7 | 15.0 |

Microheaters H_1_ and H_4_ control the ratio of the optical field amplitudes and the phase difference between the two arms of MZI_2_, respectively. The electric powers applied to H_6_, H_7_ and H_10_ are adjusted to match the coupling coefficients of MRR_1_ and MRR_2_. Microheaters H_5_ and H_8_ are used to align the resonant wavelengths of the two MRRs. The insertion loss is obtained from Fig. 5(d) at 1550 nm.

Table S2. Electric power applied to the electrodes and power variation of the first-order APF

| H_2_ (mW) | H_4_ (mW) | H_5_ (mW) | H_6_ (mW) | H_10_ (mW) | Delay (ps) | Power variation (dB) | Insertion loss (dB) |
| --- | --- | --- | --- | --- | --- | --- | --- |
| 58.8 | 68.3 | 26.6 | 0 | 25.1 | 257 | 0.91 | 2.56 |
| 60.2 | 71.3 | 25.3 | 5.5 | 25.1 | 311 | 0.73 | 3.36 |
| 61.9 | 74.5 | 24.5 | 10.5 | 25.1 | 384 | 0.80 | 6.62 |
| 62.5 | 76.0 | 2.4 | 12.4 | 25.1 | 429 | 0.80 | 8.43 |

Microheaters H_2_ and H_4_ control the ratio of the optical field amplitudes and the phase difference between the two arms of MZI_2_, respectively. The electric power applied to H_10_ is used to adjust the self-coupling coefficient between the waveguide and MRR_2_ to close to 1. The electric power applied to H_6_ is adjusted to change the coupling coefficient between the upper arm of MZI_2_ and MRR_2_. Microheater H_5_ is used to tune the resonant wavelength of MRR_1_. The insertion loss is obtained from Fig. 8(d) at 1550 nm.

References

1. C. K. Madsen and G. Lenz, Optical all-pass filters for phase response design with applications for dispersion compensation, *IEEE Photonics Technol. Lett.* **10** (1998), 994–996.
2. B. Dingel, “Multifunctional optical filter using direct-coupled and cross-coupled all-passfilters,” *IEEE Photonics Technol. Lett.* **26** (2014), 785–788.
3. W. Jiang, L. Xu and Y. Liu, Optical all-pass filter in silicon-on-insulator, *Acs Photonics* **7** (2020), 2539–2546.
4. P. Dong, W. Qian and H. Liang. Thermally tunable silicon racetrack resonators with ultralow tuning power. *Optics Express*. 18 (2010): 20298–20304.
